# Extracellular Matrix Peptides of *Artemia* Cyst Shell Participate in Protecting Encysted Embryos from Extreme Environments

**DOI:** 10.1371/journal.pone.0020187

**Published:** 2011-06-06

**Authors:** Li Dai, Dian-Fu Chen, Yu-Lei Liu, Yang Zhao, Fan Yang, Jin-Shu Yang, Wei-Jun Yang

**Affiliations:** 1 Institute of Cell Biology and Genetics, College of Life Sciences, Zijingang Campus, Zhejiang University, Hangzhou, Zhejiang, People's Republic of China; 2 Key Laboratory of Conservation Biology for Endangered Wildlife of the Ministry of Education, Hangzhou, Zhejiang, People's Republic of China; Stockholm University, Sweden

## Abstract

**Background:**

Many species of the brine shrimp *Artemia* are found in various severe environments in many parts of the world where extreme salinity, high UV radiation levels, high pH, anoxia, large temperature fluctuations, and intermittent dry conditions are often recorded. To withstand adverse environments, *Artemia* undergoes an oviparous developmental pathway to release cysts whereas, under favorable conditions, swimming nauplius larvae are formed directly via an ovoviviparous pathway. In the former case these cysts have an extraordinary ability to keep the embryos protected from the harsh environment for long periods. This is achieved through the protection by a complex out-wrapping cyst shell. However, the formation and function of the cyst shell is complex; the details remain largely unclear.

**Principal Finding:**

A shell gland-specific gene (SGEG2) was cloned and identified from a suppression subtractive hybridization library. Western blot analysis showed that SGEG2 presumably requires post-translational proteolysis in order to be processed into two mature peptides (SGEG2a and 2b). The three matrix peptides (SGEG1 reported previously, 2a, and 2b) were found to distribute throughout the cyst shell. The results of gene knockdown by RNAi and subsequent resistance to environmental stresses assays indicated that these matrix peptides are required for cyst shell formation and are involved in protecting the encysted embryos from environmental stress.

**Conclusions/Significance:**

This study revealed that extracellular matrix peptides participate in protecting embryos from extreme salinity, UV radiation, large temperature fluctuations and dry environments, thereby facilitating their survival. The cyst shell provides an excellent opportunity to link the ecological setting of an organism to the underlying physiological and biochemical processes enabling its survival. The cyst shell material has also a high potential to become an excellent new biomaterial with a high number of prospective uses due, specifically, to such biological characteristics.

## Introduction

Inland salt lakes represent one of the most hostile environments on earth in terms of their extreme salinity, high UV radiation levels, high pH, anoxia, large temperature differences, and intermittent dry conditions. Very few animals are able to survive in such an extreme environment. However, one notable exception is a small crustacean, *Artemia* [1]–[4]. In nature, to withstand adverse environments, *Artemia* undergoes an oviparous developmental pathway to release cysts. In contrast, under favorable conditions, swimming nauplius larvae are formed directly in an ovoviviparous pathway [5]. *Artemia* cyst is composed of about 4000 cells. These cells are arrested at the late gastrula stage during which nucleic acid and protein synthesis are completely turned off. This state enables embryo survival in extremely harsh environments for periods varying from several months to many years [6]. In the Free Flyer Biostack Experiment (L.D.E.F. mission), investigations have shown that *Artemia* cysts in the resting state can survive more than 5.5 years of exposure to space factors, in particular microgravity and cosmic rays [7]. Upon a favorable change in the external conditions, the embryos receive appropriate signals and proceed to hatch out as nauplii [8]. The cysts thus show extraordinary properties and provide an excellent model system for the study of biochemical adaptations to environmental extremes.

To address biochemical mechanisms of stress resistance, many studies have focused on the specific cellular molecules in the embryos. First is p26, a small heat shock/á-crystallin protein functioning as a molecular chaperone, which is reversibly trans-located into nuclei during stress and which confers thermotolerance and prevents irreversible protein denaturation [9], [10]. The second is artemin, an iron storage protein bearing structural similarity to ferritin, which has high thermal stability and functions as an RNA chaperone [11], [12]. Third not the last, there is trehalose, the non-reducing disaccharide of glucose, which protects liposomes against dehydration damage and prevents chill-damage [13], [14].

In addition to embryo's response, the shell directly contributes to cyst's extraordinary ability to resist extreme stresses. Tanguay et al have noted that the substantial tolerance of *Artemia* embryos to UV light does not result from aspects of intracellular biochemistry but rather is a result of the surrounding thick cyst shell. Such a shell, therefore, became highlighted as a biophysical adaptation of considerable importance since these embryos often receive heavy doses of UV in natural environment [15]. Similarly, Clegg reported that the cyst shell plays a critical role in desiccation tolerance, since the rate of dehydration of decapsulated cysts was found to be much higher than intact ones in his dehydration study [16]. Our previous studies have highlighted the function of the shell peptide SGEG (renamed as SGEG1 in this study), at the molecular level. We concluded that the embryo, after SGEG1 RNAi, is rendered unable to bear extreme osmotic pressure, freezing or UV irradiation exposure levels whilst embryos within intact and unaltered cysts remained relatively unaffected in identical exposure conditions [17]. However, the formation and function of the cyst shell is complex and the details remained largely unclear.

Although the cyst shells of different *Artemia* strains show substantially different surface topography in scanning electron microscopy (SEM) [18], the cyst shells of all strains exhibit the same basic structure. The cyst shell consists of a non-cellular chorion layer and a cellular embryonic cuticle layer; the former is hypochlorite-soluble and secreted by cells of the shell gland, while the latter is hypochlorite-resistant and formed by blastoderm cells [19]. In transmission electron microscopy (TEM), the non-cellular chorion layer is revealed to be composed of the thin supra cortical layer (TSCL), the cortical layer (CL) and the alveolar layer (AL) [20]. The TSCL, a wavy surface lamella that covers the outer surface of the diapause encysted embryo, probably consists of tannins or other polyphenolic compounds which may afford the hardness and strength of shell peptides, as well as the waterproofing properties of wax. The CL, tightly bound to the TSCL, has many radially-aligned aeropyles penetrating through it and may function as a channel favoring the passage of water or some volatile solutes. The AL, interior to the CL, accounts for most of the space of the chorion layer. It consists of low-density gas and may act as a float for the newly-laid cysts [21].

Biologically-formed extracellular materials such as teeth, eggshell, mollusk nacre and fish otolith differ from their inorganic counterparts in terms of the protein-mediated control of their formation, in reference to the structural properties as well as the shape and size [22]–[25]. In the case of *Artemia* cysts, although it is known that the cyst shell contains chitin, lipoprotein, haematin, and some metal elements [26], the formation of the cyst shell is complex and the details remain to be fully elucidated.

In this study, we focused on the extracellular matrix peptides which are key molecules in *Artemia* cyst shell formation and play an important role in resistance to environmental stresses. The transcripts of three extracellular matrix peptides, SGEG1, 2a and 2b are specifically expressed in the cells of shell glands. Immunolocalization analysis reveals that these matrix peptides SGEG1, 2a, and 2b are distributed throughout the cyst shell. The results of gene knockdown by RNAi and subsequent resistance assays to environmental stress indicate that these matrix peptides are required for cyst shell formation and thus participate in protecting the encysted embryos from environmental stress.

## Methods

### Culture of Artemia parthenogenetica

Cysts of *Artemia parthenogenetica* from Gahai Lake, China, were activated by soaking in saturated brine for 48 hours and freezing at −20°C for 3 months, were then incubated in 2.5% artificial seawater (Blue Starfish, Zhejiang, China) at 28°C with continuous light following hydration at 4°C for 5 hours. Swimming nauplii were reared in 8% (w/v) artificial seawater under LD (light: dark cycles) of 4 (12:00 − 16:00): 20 hours for the oviparous *Artemia* or in 4% (w/v) artificial seawater under LD 16 (7:00 – 23:00): 8 for the ovoviviparous *Artemia*. The water temperature was kept at 28°C. The water was supplemented with Chlorella powder (Fuqing King Dnarmsa Spirulina Co. Ltd., Zhejiang, China) as brine shrimp food.

### Sequence validation and analysis

Suppression subtractive hybridization (SSH) was performed as described by Liu et al [17]. A full-length SGEG2 cDNA was acquired by concatenating two clones from the SSH library. A complete fragment of the SGEG2 cDNA was then amplified by the gene-specific primers 2F and 2R (Table S1) to confirm they originated from a single transcript. The sequenced cDNA was edited and analyzed using Lasergene 7 (DNAStar Inc., USA), and the deduced amino acid sequence of the peptide was predicted using the websites PredictProtein (http://www.predictprotein.org/) and Scratch Protein Predictor (http://www.ics.uci.edu/baldig/scratch/). The Blast (both blastx and blastn) search was performed against the NCBI website (http://www.ncbi.nlm.nih.gov/blast). The nucleotide sequence of SGEG2 was submitted to GenBank/DDBJ/EMBL under the accession number GU945200.

### Northern blot analysis

A DIG-labeled cDNA fragment (925 bp) amplified by the primers 2F and 2R (Table S1) was used as a probe for the Northern blot analyses. The total RNA of both oviparous and ovoviviparous *Artemia* was extracted using Trizol Reagent (Invitrogen). All samples (10 ìg for each tissue) were separated by agarose gel electrophoresis, then transferred to a nylon membrane (Hybond-N, Amersham) for overnight hybridization at 42°C, washed twice at 55°C, and finally detection was performed using the DIG chemiluminescent detection system (Roche).

### In situ hybridization (ISH)

The DIG-labeled sense and antisense RNA probes, corresponding to nucleotides 112−408 of SGEG2 cDNA, were amplified (primed by iF and iR, Table S1) and cloned into the plasmid vector pSPT18 at the EcoRI and XbaI sites. They were then transcribed in vitro from the EcoRI- and XbaI- linearized templates, respectively (DIG RNA Labeling kit SP6/T7, Roche). For tissue section preparation, *Artemia* were placed in an ice-bath for 1−2 min until they were lightly anesthetized, snap-frozen in liquid nitrogen, and embedded in Tissue-Tek™ (Sakura Finetechnical Co. Ltd.). Then, 8-ìm-thick frozen sections were prepared using a frozen ultramicrotome. The sections were air dried, fixed with paraformaldehyde, digested with Proteinase K and hybridized at 42°C overnight. These sections were washed at 52°C and blocked using blocking solution (Roche). They were then treated with anti-DIG-AP conjugate (1∶500, Roche), and visualized using the colorimetric substrates nitroblue tetrazolium/5-bromo-4-chloro-3-indolylphosphate (NBT/BCIP, Promega) according to the manufacturer's instructions. Finally, photographs were taken on an inverted microscope (ECLIPSE TE2000-S, Nikon).

### Western blot analysis

The anti-SGEG1 antibody was as used in the previous study [17], and the anti-SGEG2a and 2b antibodies were raised in rabbit (HuaAn Biotechnology Co. LTD., Zhejiang, China) against peptides containing the partial amino acid sequences (CQSKTFYDNDKDLAGPY and CKPRSQESGQRVQKD, respectively) that represented the best epitopes. Each sample was homogenized in the loading buffer (125 mM Tris, 4% sodium dodecyl sulfate (SDS), 5% 2-mercaptoethanol, 20% glycerol, 0.02−0.04% bromophenol blue, pH 6.8) and boiled for 10 min. After centrifugation at 12,000 g for 10 min at 4°C to remove insoluble shell fragments, supernatants were quantitatively measured using the direct UV method. Fifty micrograms of protein from each sample were separated on 12.5% or 15% SDS-polyacrylamide gel electrophoresis (SDS-PAGE) gels and transferred to PVDF membranes (Millipore). The membranes were incubated with anti-SGEG1 antibody (1∶500), anti-SGEG2a antibody (1∶2000), anti-SGEG2b antibody (1∶2000), and anti-á-tubulin antibody (1∶50000) (Beyotime, China) overnight at 4°C, and detection was performed using the BM Chemiluminescence Western Blotting Kit (Roche).

### Immunohistochemistry

Cysts were soaked in ice-cold 2.0 M sucrose for 20 min, and the cyst walls were nicked. Cysts were then fixed in 4% paraformaldehyde and 0.2% glutaraldehyde overnight at 4°C. To obtain ultrathin sections, these cysts were embedded in 12% gelatin in 1 M PBS at 37°C for 30 min, and transformed to 4°C until solidification. Cysts in gelatin were then cut into nubs of approximately 0.1×0.1 cm^2^. These nubs were sequentially immersed in 30%, 50% and 70% (w/v) sucrose solution while sedimenting. Sections of 70 nm were cut using a microtome (Leica EM UC6), incubated with the anti-SGEG1 antibody (1∶100), anti-SGEG2a antibody (1∶500) or anti-SGEG2b antibody (1∶500), and coated with 15 nm gold nanoparticle-conjugated secondary antibody (1∶30, Sigma-Aldrich). Cysts were viewed under the TEM (JEM-1230) and photographed at a voltage of 80 kV.

### RNA interference

Sequence-specific primers (CExpF/CRiR, iF/iR, and green fluorescent protein (GFP) F/R, Table S1) were designed from the SGEG1, SGEG2, and GFP (pcDNA3.1/NT-GFP-Topo, Invitrogen) cDNA sequence. The 361 bp SGEG1, 297 bp SGEG2, and 359 bp GFP (as the negative control) cDNA fragments were subcloned into the plasmid pET-T7 [27] at the XbaI and EcoRI sites for SGEG2 and GFP, and XbaI and BamHI sites for SGEG1, because of the EcoRI site located at positions 72−77 of SCEG1 cDNA. The recombinant plasmids were transformed into Escherichia coli HT115 cells, and then the double-stranded (ds) RNAs were produced and purified as described by Yodmuang et al [28]. Based on the results for the basic and quantitative controls (Fig. S2), four preparations of 300 ng SGEG1, 100 ng SGEG2, 300 ng SGEG1 plus 100 ng SGEG2, and 300 ng control dsRNA of GFP, were injected into the body cavity of *Artemia* just before ovarian development. For each of the four preparation types, 500 individual *Artemia* were injected. An UltraMicroPump II equipped with the Micro4™ MicroSyringe Pump Controller was used for the microinjection. The RNAi and control *Artemia* were cultured in 8% artificial seawater under the condition of LD 4∶20. The RNAi-treated and control cysts were observed by light microscopy and collected for the following experiments.

### Real-time PCR

Total RNAs were extracted from ovisacs (middle stage of the cyst-destined adults: lateral pouches filled with oocytes) of RNAi-treated and control *Artemia* 6 days after injection. After reverse transcription, all real-time PCR reactions were performed on the Bio-Rad MiniOpticonTM Real-Time PCR System using the SYBR® Premix Ex TaqTM (TaKaRa Bio Inc.) and 200 nM SGEG1- and SGEG2-specific primers (CQF/CQR and rtF/rtR, Table S1). Cycling parameters were: 40 cycles of 10 s at 95°C (30 s only for the first cycle), 10 s at 56°C, and 10 s at 72°C (5 min only for the last cycle). Dissociation curves were analyzed at the end of each run to determine the purity of the product and specificity of amplification. Relative transcript levels are presented as fold-changes calculated using the comparative CT method as described by Livak and Schmittgen [29], [30] with 18S cDNA (amplified by the primers 18S F/18S R in Table S1) as the internal reference. All data are given as means ± SEM of independent experiments from three separate RNA pools. All statistical analyses were performed using one-way analysis of variance (ANOVA), and the difference was considered significant for p<0.01.

### TEM and SEM analysis

A TEM study was carried out following the method described by Hofmann and Hand [31]. Cysts were soaked in ice-cold 2.0 M sucrose for 20 min to reduce the internal turgor pressure of the embryo, thereby preventing extrusion and damage of tissue. Consequently, cyst walls were nicked and fixed in 2.5% glutaraldehyde prepared in 3% NaCl for 12 hours. They were then washed and post-fixed in 1% osmium tetroxide in 3% NaCl solution, dehydrated in a graded acetone series, and embedded in spurr resin. Sections of 70 nm were cut with a microtome (UC6, Leica), stained with 2% uranyl acetate and Reynold's solution (0.2% sodium citrate and 0.2% lead nitrate), then viewed under a TEM (JEM-1230, JEOL), and photographed at a voltage of 70 kV. For SEM analysis, the RNAi-treated and control cysts were each fixed in 2.5% glutaraldehyde for 2 hours. These cysts were washed and dehydrated in a graded acetone series, and critical point dried with CO_2_ using a critical point dryer (Hitachi HCP-2). These were then used for sputter coating with gold (Hitachi E-1010) for SEM (Hitachi S-3000N).

### Assay of resistance to environmental stresses for RNAi-treated cysts

RNAi-treated and control cysts were collected for the resistance to environmental stress test, which includes extreme osmotic pressure, high temperature, freezing, organic solvent, and UV irradiation. Triplicate experiments were performed for each of these physiological stresses.

For the test of coping with extreme osmotic pressure, the RNAi-treated and control cysts were soaked in 6 M NaCl for 4 hours or in deionized water for 16 hours. After the above tests, the SGEG1 and double RNAi-treated cysts were incubated in 3% artificial sea water with continual illumination at 25°C for hatching. The hatching rates were then investigated for a period of 72 hours. In contrast, the SGEG2 RNAi-treated and control cysts were activated before hatching by soaking in saturated seawater for 48 hours and freezing at −20°C for 3 months.

For the heat shock and freezing stress test, the SGEG1 and double RNAi-treated cysts, the activated SGEG2 RNAi-treated cysts and the control cysts were incubated in a water bath at 50°C for 5 min, or frozen at −20°C, then incubated in 3% artificial sea water for investigation of the hatching rates.

For the test of resistance to organic solvent, the SGEG1-treated, double RNAi-treated, and activated SGEG2 RNAi-treated, as well as control cysts, were incubated in 3% artificial sea water with 0.5 M methanol for investigation of hatching rates.

For the UV irradiation test, the SGEG1 RNAi-treated, double RNAi-treated, activated SGEG2 RNAi-treated and control cysts were exposed directly to UV irradiation (310 nm) at 3.6 joules/cm^2^. Partial cysts were then incubated in 3% artificial sea water for investigation of hatching rates. Partial cysts were used to perform the neutral comet assay. All steps were carried out on ice or in the cold to minimize repair processes according to the manufacturer's instructions (Trevigen). For each data point, two to three areas with 50 cells each on parallel slides were scored and DNA in tail (%) was calculated for each cell using the image-analysis software CASP [32]. The median of DNA in tail (%) was calculated for each area and the presented values are the means of the medians of each data point. At least two independent experiments were performed. The t-test was used to determine statistically-significant differences between normal and irradiated cells.

## Results

### Three extracellular matrix peptides encoded by two novel genes are the shell gland-specific genes of cyst-destined *Artemia*

**Figure 1:**
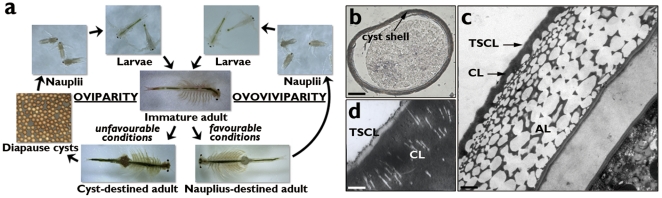
Two developmental pathways of *Artemia* and structure of the cyst shell. a, Two developmental pathways of *Artemia*. b–d, The structure of the cyst shell. Inverted microscope at ×400 magnification, scale bar, 30 µm (b). TEM at ×8,000 magnification, scale bar, 1.5 µm (c). TEM at ×30,000 magnification, scale bar, 0.2 µm (d). TSCL, thin supra cortical layer; CL, cortical layer; AL, alveolar layer.

In our laboratory culture system of *Artemia parthenogenetica* (Fig. 1a), the oviparous life cycle is maintained in 8% (w/v) artificial seawater under LD 4∶20, while the ovoviviparous mode is sustained in 4% artificial seawater under LD 16∶8. In order to understand the formation and function of the cyst shell, a suppression subtractive hybridization library of *A. parthenogenetica* was constructed in which double-stranded cDNA (dscDNA) from oviparous and ovoviviparous ovisacs were used as tester and driver, respectively. Two shell gland-specific genes were identified from the library, and their amino acid sequences were conceptually translated. One of these, SGEG1, has been reported previously [17]. Another, SGEG2, encoded by a 975 bp complete cDNA sequence, consists of a 20 amino acid putative signal peptide and a 255 amino acid putative pre-peptide which may be processed into two mature peptides SGEG2a and 2b, presumably at a kexin 2 cleavage site (Arg-Tyr-Lys-Arg) in the 110−113 position (Fig. S1). These genes and their encoded peptides share no apparent homology with any other known genes or proteins in the DDBJ/EMBL/GenBank database.

**Figure 2:**
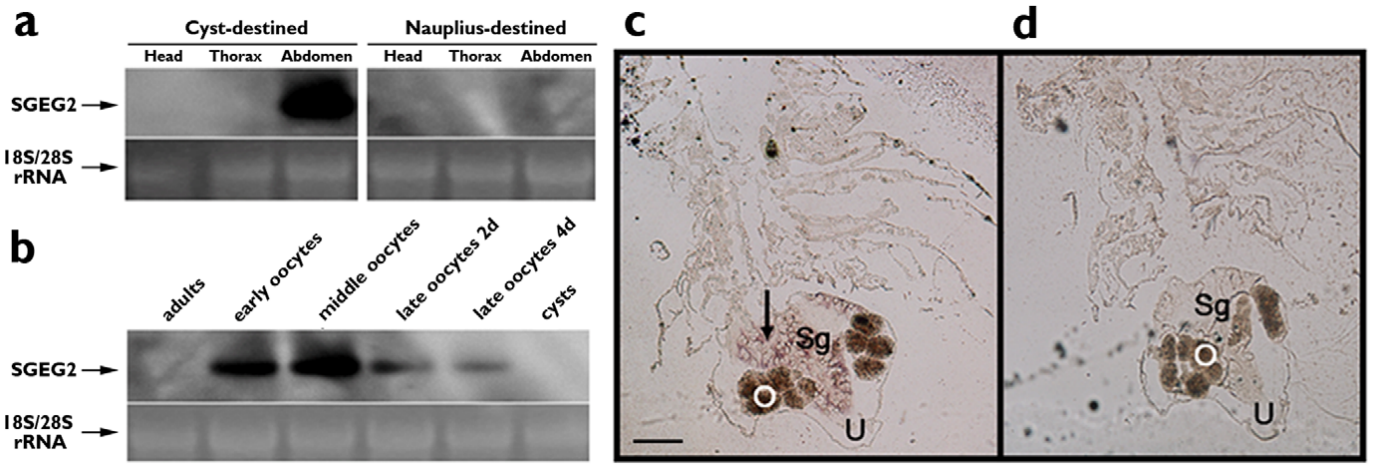
The characteristic and localization of SGEG2 mRNA. (a) Northern blot analysis of SGEG2 in various tissues of *A. parthenogenetica*. (b) Northern blot analysis in various oviparous developmental stages. Adults, cyst-destined female adults without oocytes; early oocytes, with the oocytes in the ovaries; middle oocytes, with the oocytes in the lateral pouches; late oocytes, with the oocytes in the uterus for 2 days and 4 days. The ethidium bromide-stained 28S/18S rRNA bands were used as a control for loading variation. c, d, The localization SGEG2 mRNA in the ovisac of oviparous *Artemia*. The 297 bp DIG-labelled sense and antisense RNA probes were used for in situ hybridization. The distribution of the positive brown signals in cells of shell glands are indicated by black arrow (c). Control (d) was with sense RNA probe (cross section ×40). U, uterus; Sg, shell gland; O, oocyte. Scale bar, 200 µm.

In a case similar to that of SGEG1 [17], Northern blot analysis revealed that SGEG2 mRNA was exclusively expressed in the abdomen of oviparous *Artemia* and was markedly accumulated during cyst shell formation (Fig. 2a and 2b). The shell glands consist of several clusters of gland cells [33]. ISH showed that SGEG2 mRNA was expressed only in the secreting cells of the shell glands (Fig. 2c). In contrast, the signal was absent when hybridization was performed using a sense control probe (Fig. 2d).

### The matrix peptides SGEG1, 2a, and 2b, are distributed throughout the cyst shell

**Figure 3:**
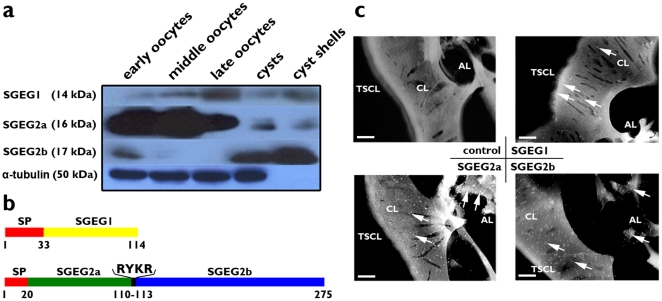
Expression and localization of the SGEG1, 2a and 2b. a, Western blotting analysis. Western blots were probed with the indicated antibodies. α-tubulin served as the internal standard. 50 µg of proteins were loaded for each lane. b, Schematic representation of deduced amino acid sequences of SGEG1, 2a and 2b. The red boxes are putative signal peptides. The yellow box is SGEG1 peptide. The green box is SGEG2a peptide and blue is SGEG2b peptide. c, Immunohistochemical localization in TEM image analysis using gold nanoparticles at ×20,000 magnification. No primary antibody was added in the control. The slices of cyst shell incubated with antibody to SGEG1, 2a and 2b. Arrows indicate the gold nanoparticles. Scale bars, 0.25 µm.

Using polyclonal antibodies specific against SGEG1, 2a and 2b, three positive bands of 14 kDa, 16 kDa and 17 kDa were identified in the extracts of shell gland-containing ovisac and cysts (Fig. 3a). Furthermore, Western blot showed that SGEG2was processed into two mature peptides 2a and 2b (Fig. 3a and 3b). It is predicted that SGEG2a contains three alpha-helix regions (comprising residues 24–25, 31–34, and 51–69) and one beta-sheet region (comprising residues 40–47); Putative N-glycosylation sites Asn^49^, phosphorylation sites Tyr^74^ and Tyr^82^ are predicted in SGEG2a. SGEG2b contains five alpha-helix regions (comprising residues 117–122, 130–133, 190–208, 211–247 and 256–263) and five beta-sheet regions (comprising residues 135–140, 154–174, 182–185, 252–255 and 267–270); Putative N-glycosylation sites Ser^114^, Tyr^126^, Ser^135^, Tyr^143^, Arg^154^, Ser^188^ and Ser^267^, phosphorylation sites Asn^124^, Asn^180^ and Asn^255^ are predicted in SGEG2b (http://www.ics.uci.edu/baldig/scratch/ and http://www.predictprotein.org). However, their tertiary structure could not be predicted exactly, due to there being no known structurally similar sequence with which to construct a prediction model.

To accurately determine the distribution of the three peptides in the cyst shell, immunohistochemical localization in combination with TEM was performed. In contrast to the control, after incubation with antibody against SGEG1, gold nanoparticles were observed with back-scattered electron imaging in the TSCL and CL of the cyst shell. Further, a large number of gold nanoparticles were observed in the CL and AL of the cyst shell after incubation with antibodies against SGEG2a and 2b (Fig. 3c). None of SGEG1, 2a or 2b could be detected in the embryonic cuticle and inner embryos (data not shown). Because the chorion layer was easy to detach from the embryonic cuticle and the inner embryo during the sections preparation, we just intercepted the images containing the chorion layer.

### SGEG1, 2a and 2b are required for cyst shell formation

**Figure 4:**
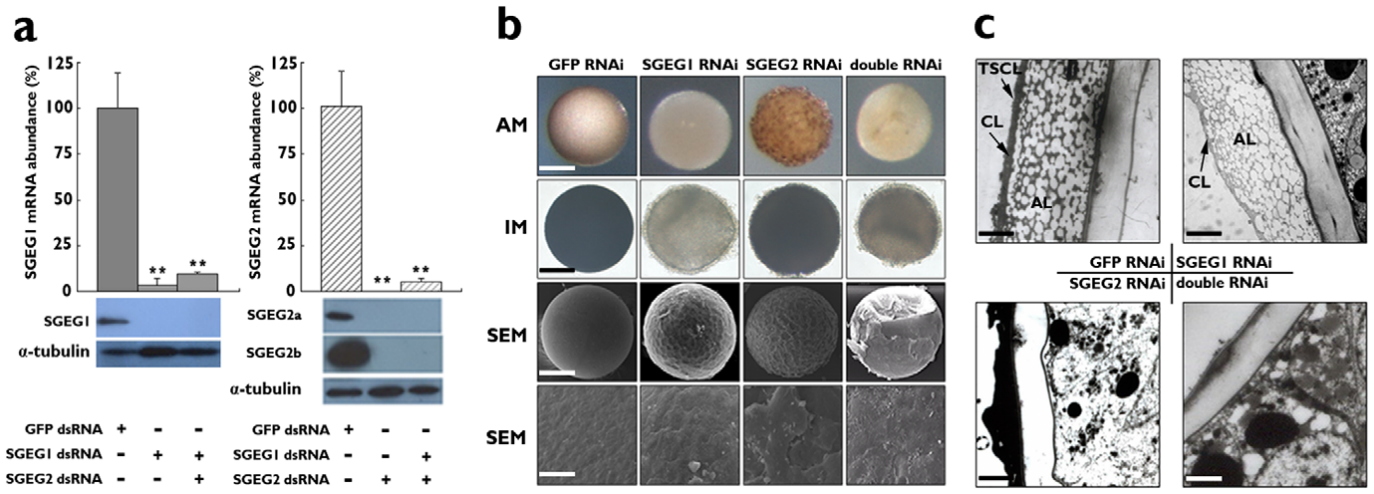
RNAi treatment and phenotypes of cysts. a, The expression levels of SGEG1 and 2 mRNA were determined with real-time quantitative PCR after RNAi treatments. The expression levels of the GFP dsRNA injected group are attributed a relative value of 100 as a control. Plotted is the mean ± s.d (n = 10, ** P<0.001, one-tailed t-test). The peptides in cysts were analyzed by Western blotting and alpha-tublin was used for loading control. b, c, The phenotypes of all RNAi-treated cysts. b, Observation by anatomical microscopy (AM) at ×80 magnification, scale bar, 100 µm; Inverted microscopy (IM) at ×100 magnification, scale bar, 80 µm; SEM at ×400 magnification, scale bar, 60 µm; and SEM at ×2,000 magnification (the lower panel) , scale bar, 12 µm. c, Observation by TEM at ×8,000 magnification, scale bars, 3 µm.

To clarify the structural roles of these peptides in cyst shell formation, knockdown of SGEG1 or 2, or both, by means of RNAi was performed. The dsRNAs, designed from the cDNA sequences, were injected into the body cavity of oviparous *Artemia* just before ovarian development. SGEG1 and 2 expression levels, measured 5 days after oocyte development, decreased with increasing injection dose of the respective dsRNAs (Fig. S2). Following injection with 300 ng of dsRNA for SGEG1, 100 ng dsRNA for SGEG2, and both of these together, the levels of these mRNAs were reduced to less than 10% of those of the GFP dsRNA-injected group, and expression of the peptides was decreased to levels so low that they could not be detected by Western blot analysis (Fig. 4a).

The cysts of all RNAi-treated groups exhibited significantly abnormal phenotypes. It was observed that the normal cysts were floating on the surface of 8% seawater and were adhered to the wall of the culture vessel, but all of the RNAi-treated cysts sank to the bottom; the surface of SGEG1 and double RNAi-treated cysts turned soft, but the SGEG2 RNAi-treated cysts possessed the rigid surface as the control group. Using anatomical and inverted microscopy, it was observed that in contrast to the control cyst shell, the SGEG1 RNAi-treated cysts became transparent and their surface was rough. The SGEG2 RNAi-treated cysts were more fuscous and opaque, and their surface was more uneven. Moreover, the double RNAi-treated cysts were shrunken and transparent, and the surface adhesive and coarse (Fig. 4b). Using SEM, it was found that instead of exhibiting the compact and smooth shell of the control cyst, the cyst shell in the SGEG1 and SGEG2 RNAi-treated groups was lamellar and rough, and the shell was particularly flimsy and rough after double RNAi-treatment (Fig. 4b). In the shell of SGEG1 RNAi-treated cysts, observations using TEM revealed that the TSCL had disappeared and the CL was less than half the thickness of the control CL. After SGEG2 RNAi treatment, the AL had disappeared completely and the outer layer of the TSCL and CL was irregular. In the case of double RNAi treatment, the chorion layer, including the TSCL, CL and AL, had largely disappeared (Fig. 4c).

### SGEG 1, 2a and 2b participate in protecting the *Artemia* embryo from environmental stress

**Figure 5:**
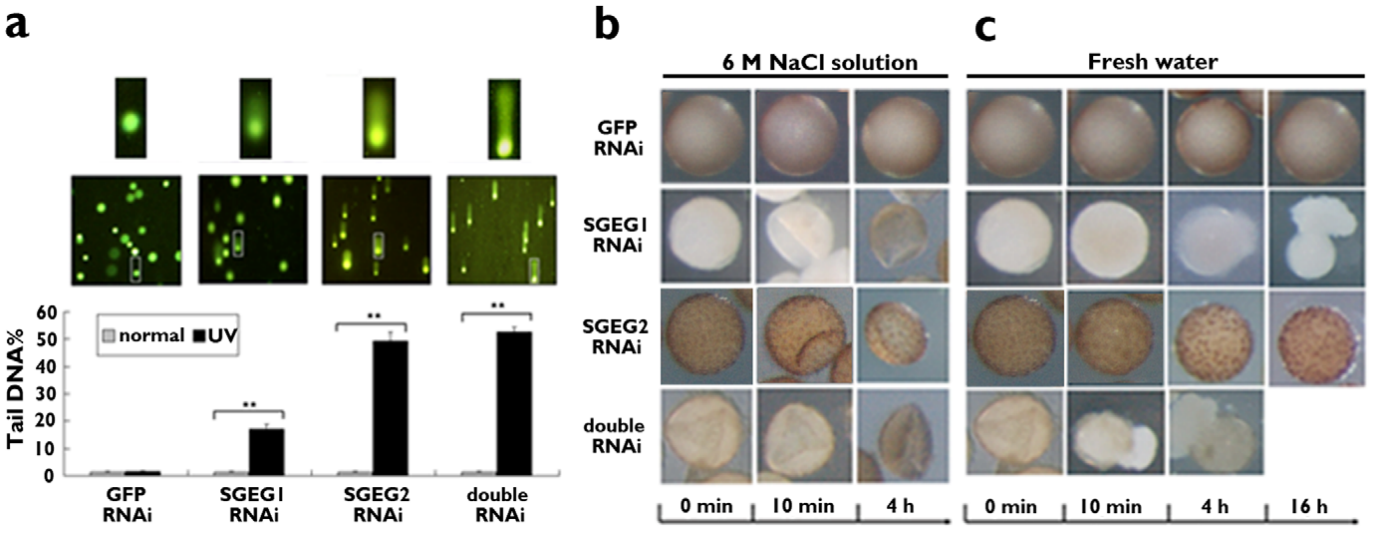
Assay of resistance of the cysts to UV radiation and osmotic stress. a, Comet assay. DNA damage in the cells of all RNAi-treated cysts after UV irradiation with 3.6 joules/cm^2^ for 30 min. Damage is expressed as DNA in tail (%). Plotted is the mean ± s.d (n = 50, ** P<0.001, one-tailed t-test). b, c, The cysts were incubated in 6 M NaCl solution (b) and in fresh water (c).

**Figure 6:**
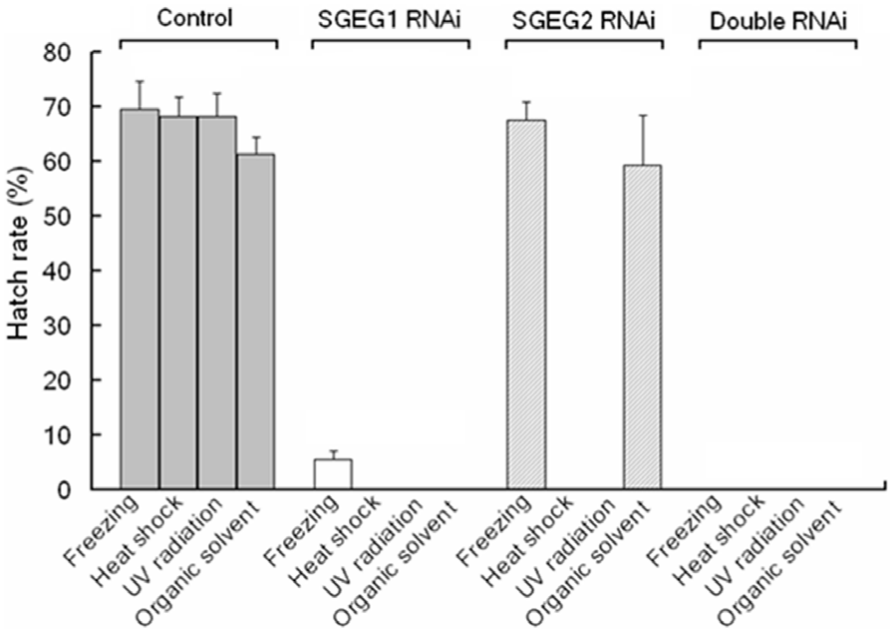
Resistance of encysted embryos to environmental stress of freezing, heat shock, UV radiation, and organic solvent. Triplicate experiments were performed for each of these physiological stresses. In every group, 100 cysts were taken to hatch; the hatch rate was calculated by the number of live nauplius resulting.

In order to clarify the involvement of these extracellular matrix peptides in protective functions of the cyst shell, we exposed RNAi-treated and control cysts to UV radiation at 3.6 joules/cm^2^. A single-cell gel electrophoresis assay (also known as a comet assay) was performed to assess the DNA damage induced by UV radiation in the cells of RNAi-treated cysts. As shown in Fig. 5a, a significantly greater DNA tail length and intensity were observed in the cells of all RNAi-treated cysts after UV radiation exposure. Furthermore, the DNA damage was markedly more severe in SGEG2 RNAi- and double RNAi-treated cells than in SGEG1 RNAi-treated cells. All RNAi-treated cysts were unable to survive and hatch after just 30 minutes of exposure, while control cysts were able to hatch normally (Fig. 6).

The cysts of *Artemia* show resistance to osmotic pressure, large temperature fluctuations, UV radiation, and other environmental stresses [15]. In our experiments, this ability was highly compromised in cysts following RNAi-treatments. Fig. 5b showed that all the RNAi-treated cysts shrank drastically after 10 min in 6 M NaCl solution, while the control cysts retained their round shape. However, only the SGEG2 RNAi-treated cysts can restore the original round shape after immersion in the solution which was lower than normal osmotic pressure (data not shown). In fresh water (Fig. 5c), the SGEG1 RNAi-treated cysts burst and the content of the embryos began leaking out just after 4 h immersion, with the full content of the cysts being almost totally lost by 16 h immersion. For the double RNAi-treated cysts this process was much faster, with bursting occurring after just 10 min immersion and the content of the embryos inside having leaked out completely after 4 h immersion. In comparison, the control and SGEG2 RNAi-treated cysts remained completely intact. In addition, the cysts in the three RNAi-treated groups were not able to survive after heat shock at 50°C for 5 minutes. Futhermore, the SGEG1 and double RNAi-treated cysts were not able to withstand 3 days frozen at −30°C or treatment with an organic solvent (0.5 M methanol) (Fig. 6).

## Discussion

To withstand adverse environments and ensure species survival, *Artemia* undergoes oviparous development to release the encysted embryo. In contrast, under favorable conditions swimming nauplius are formed directly via an ovoviviparous pathway (Fig. 1a). The diapause embryo is encapsulated in a complicated non-cellular shell (Fig. 1b–d) which shields the embryo from extremely environmental stress. The shell morphology of *Artemia* cysts has been studied intensively [15], [16], but little is known regarding the molecular mechanisms of shell formation. Recently, we reported that a peptide (SGEG1) secreted by the shell gland protects the encysted embryo from some adverse environments [17]. In the present study, another peptide (SGEG2) was identified and our findings suggest that it can be processed into two mature peptides, SGEG2a and 2b. Using RNAi, immunohistochemistry and protective assays against environmental stress indicated that SGEG1, 2a and 2b are required for cyst shell formation and participate in protecting the embryos from the variations of extreme environments. These results represent a significant breakthrough in research on the *Artemia* cyst shell at the molecular level.

The expression patterns of SGEG1 and 2 transcripts are very similar, with both accumulating when oocytes are in the lateral pouches. However, their protein expression profiles are greatly different. SGEG1, 2a and 2b are absent in the inner embryos and are specifically expressed in cyst shell, but SGEG1 is scattered throughout the outer region of the cyst shell, whereas large amounts of SGEG2a and 2b are located in the inner region of the cyst shell. Interestingly, even if SGEG2a and 2b are post-translational processing products from the same pre-peptide, SGEG2a is expressed at higher levels at different stages of oocytogenesis, while SGEG2b is expressed at higher levels in the cyst. This difference reflects the different contributions of SGEG2a and SGEG2b to shell formation.

*Artemia* diapause embryos are produced in ovaries, and then pass through oviducts and lateral pouches, finally converge into uterus, where the different regions of the shell are orderly assembled prior to cysts release. SGEG1 is distributed in the outer chorion layer, whereas SGEG2a and 2b in the inner chorion layer of the shell. Therefore, just after SGEG2a and 2b are assembled perfectly, SGEG1 can be assembled correctly. From the TEM images, in the SGEG1 RNAi-treated cysts, the remaining architecture of the shell is similar to that of the control cyst; however, in SGEG2 RNAi-treated cysts, due to the absence of the inner structure, the outer layer of the shell cannot be assembled perfectly, so it looks uneven and irregular. Although there is no evidence suggesting a direct functional interaction between SGEG1 and 2 in the present study, they may directly or indirectly interact, based on their nested distributions in the cyst shell.

Normal cysts have a very smooth external surface. Cyst shell consists of the inner embryonic cuticle and the outer chorion layer with the tertiary structure: TSCL, CL and AL from out to inner in TEM; the aeropyles penetrate through the mid area of CL. All RNAi-treated cysts seem uneven and incompact; TEM images suggest SGEG1 RNAi-treated cysts lost TSCL and most of CL; AL is disappeared in SGEG2 RNAi-treated cysts; entire chorion layer is disappeared after both SGEG1 and 2 genes knockdown.

Normal cysts released from uterus float on the surface of 8% seawater. All RNAi-treated cysts sank on the bottom. Sugumar and Munuswamy considered AL is filled a lot of gas or low-density liquid which can maintain the buoyancy for the newly laid cysts [21]. Therefore, SGEG2 RNAi-treated cysts just sank on the bottom because of AL missing. Because the absence of TSCL and most of CL results in brine water filling in AL, SGEG1 RNAi-treated cysts also sank on the bottom. The chorion layer is disappeared in double RNAi-treated cysts which can't maintain the buoyancy obviously.

In SGEG1 and double RNAi-treated cysts, the existed embryonic cuticle as biological membrane has the characteristic of ionic selectivity and allows substances with low molecular weight pass freely, but extreme ionic concentration and organic solvents beyond the limits of tolerance can result in embryonic death. This may explain why SGEG1 and double RNAi-treated cysts are unable to resist extreme osmotic pressures and organic solvent. In contrast, normal cysts and SGEG2 RNAi-treated cysts were able to resist such stresses. The aeropyles penetration into the mid area of the CL may mediate cysts resistance to extreme osmotic stress and organic solvents. We considered that the aeropyles not only functions as a channel favoring the passage of water or some volatile solutes such as methanol [21], but more importantly, that the aeropyles limit the water quantity or methanol saturation.

After SGEG1 gene knockdown, Liu et al detected differences in metallic elemental compositions and found that iron level sharply decreased compared with control cysts. Hematin, a light-screening iron-binding hemopigment, may be located in the same region as SGEG1 in the cyst shell [17]. The UV resistance defect in SGEG1 RNAi-treated cysts may be due to the decrease of hematin. The comet assay showed SGEG2 RNAi-treated cysts were injured more seriously than SGEG1 RNAi-treated cysts in response to UV irradiation. The outer surface curvature of large pores of AL region, which is absent in SGEG2 RNAi-treated cysts, may act to filter out or reflect UV radiation.

Normal cyst can resist large temperature fluctuations, ranging from −30°C to 50°C, over a long period. SGEG2 RNAi-treated cysts can endure frozen no less than three months, but SGEG1 RNAi-treated cysts can resist the same stress no more than three days. Dense and opaque TSCL and CL may play important role on defending cold. All the RNAi-treated cysts can't survival in high temperature test; therefore, intact chorion layer is indispensible for heat resistance.

SGEG1, 2a and 2b are localized in cyst shell specifically. It is known that the *Artemia* cyst shell contains lipoprotein, chitin, haematin, and some metal elements [26] as well as the organic extracellular molecules SGEG1, 2a and 2b. The cyst shell is thus considered to be constructed via a complex process which includes the self-assembly of other inorganic components and the peptides. The interaction between the three peptides, SGEG1, 2a and 2b and other compositions may play an important role in formation of the *Artemia* cyst shell. After knockdown both SGEG1 and 2 genes, the cysts lost the chorion layer completely can't resist any environmental stress. Therefore, the shell exposed to the environments is a natural and direct barrier to protect the embryos from extreme environmental stresses.

### Conclusions

These results indicate that the cyst shell plays an important role in protecting *Artemia* embryos from various environmental stresses, thereby facilitating their survival. Further, the role of the three matrix peptides in cyst shell morphogenesis represents an interesting example of biological influence in the process of formation of extracellular materials which show remarkable properties. Thus, the cyst shell material has great potential to become an excellent new biomaterial with a high number of prospective uses due, specifically, to such biological characteristics.

## Supporting Information

Figure S1 **Nucleotide and deduced amino acid sequences of the cDNA encoding SGEG2.** The nucleotide and amino acid residue numbers are indicated on the left. The start (ATG) and stop codons (TAA) are in bold. The putative signal peptides are shaded, and the kexin 2 cleavage site (RYKR) is boxed. The asterisk denotes termination of amino acids. The putative polyadenylation signal (AATAAC) is underlined.
(TIF)

Figure S2 **Knockdown of the SCEG1 and 2 genes by means of dose-response RNAi.** a, b, The expression levels of SGEG1 (a) and SGEG2 (b) mRNA in shell glands of *Artemia* were determined by real-time PCR 5 days after the cysts formed. Intact: no injection; control: GFP dsRNA injected group. The SGEG1 and SGEG2 mRNA expression levels of the control group were assigned a relative value of 100%. Thirty *Artemia* individuals were used in each experiment. All data are given as means ± SEM. The asterisks indicate a highly significant difference (p<0.01) between the test and control groups as analyzed by one-way ANOVA. Representative cysts of all treatments are shown in the lower panel.
(TIF)

Table S1 **Nucleotide sequences and positions of primers used in PCR.**
(DOC)
