# Supplementary figures and images for: Extracellular Matrix Peptides of Artemia Cyst Shell Participate in Protecting Encysted Embryos from Extreme Environments
Source: PLoS One. 2011 Jun 6;6(6):e20187. doi: 10.1371/journal.pone.0020187 (PMC3108945; doi:10.1371/journal.pone.0020187)

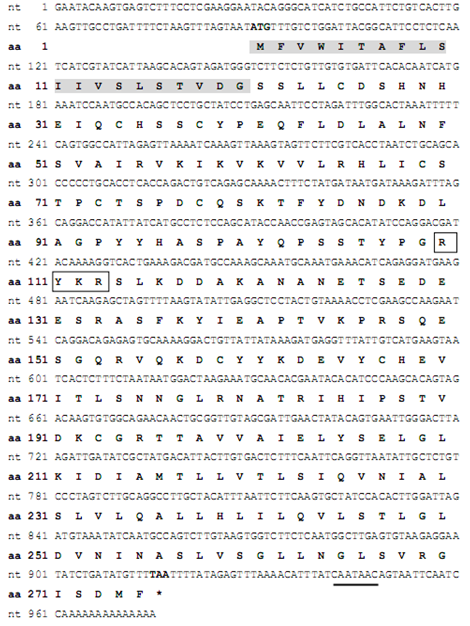

Supplement: Figure S1 — Nucleotide and deduced amino acid sequences of the cDNA encoding SGEG2. The nucleotide and amino acid residue numbers are indicated on the left. The start (ATG) and stop codons (TAA) are in bold. The putative signal peptides are shaded, and the kexin 2 cleavage site (RYKR) is boxed. The asterisk denotes termination of amino acids. The putative polyadenylation signal (AATAAC) is underlined. (TIF) [file pone.0020187.s001.tif]

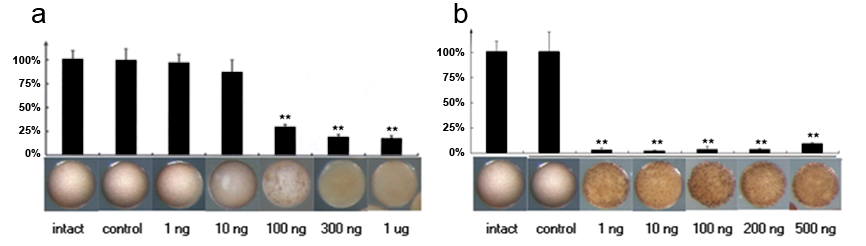

Supplement: Figure S2 — Knockdown of the SCEG1 and 2 genes by means of dose-response RNAi. a, b, The expression levels of SGEG1 (a) and SGEG2 (b) mRNA in shell glands of Artemia were determined by real-time PCR 5 days after the cysts formed. Intact: no injection; control: GFP dsRNA injected group. The SGEG1 and SGEG2 mRNA expression levels of the control group were assigned a relative value of 100%. Thirty Artemia individuals were used in each experiment. All data are given as means ± SEM. The asterisks indicate a highly significant difference (p<0.01) between the test and control groups as analyzed by one-way ANOVA. Representative cysts of all treatments are shown in the lower panel. (TIF) [file pone.0020187.s002.tif]
