# Supplementary material for: Extracellular Matrix Peptides of Artemia Cyst Shell Participate in Protecting Encysted Embryos from Extreme Environments
Source: PLoS One. 2011 Jun 6;6(6):e20187. doi: 10.1371/journal.pone.0020187 (PMC3108945; doi:10.1371/journal.pone.0020187)
Supplement: Table S1 — Nucleotide sequences and positions of primers used in PCR. (DOC) [file pone.0020187.s003.doc]

**Supplementary Table 1** Nucleotide sequences and positions of primers used in PCR.

| Primer | Length (bp) | Position | Direction^*^ | Sequence (5'-3') |
| --- | --- | --- | --- | --- |
| 2F | 22 | 39-60 | F | TCATCTGCCATTCTGTCACTTG |
| 2R | 22 | 942-963 | R | TTGGATTGAATTACTGTTATTG |
| iF | 25 | 112-128 | F | GCTCTAGATCCTCTCAATCATCGTA |
| iR | 25 | 391-408 | R | GGAATTCATGTGCTACTCGGTTGGT |
| rtF | 24 | 503-526 | F | TATTGAGGCTCCTACTGTAAAACC |
| rtR | 24 | 678-701 | R | TAGTTCAATCGCTACAACCGCAGT |
| CExpF | 30 |  | F | CGCGGATCCATGGGGGTAAAGGAAGTTTTG |
| CRiR | 30 |  | R | GCTCTAGACTAAATTTGCATCTGTTTAATCC |
| CQF | 20 |  | F | CGCGGATCCATGGGGGTAAAGGAAGTTTTG |
| CQR | 20 |  | R | CTAAGTTGGTGTCCGTCAAA |
| 18sF | 22 |  | F | GAAGCACTCTCTACCCTTCCTG |
| 18sR | 25 |  | R | ATTCACACGTAGAAAATATACATCG |
| GFPF | 30 |  | F | GGAATTCAACTTACCCTTAATTTTATTTGC |
| GFPR | 28 |  | R | GCTCTAGAGCCATTCTTTGGTTTGTCTC |

^*^ F and R indicate the forward and reverse directions, respectively. The underlined regions represent the adscititious recognition sequences of restriction endonucleases.
